# Effectiveness of Photobiomodulation as a Treatment for Xerostomia in Patients with Head and Neck Cancer Undergoing Radiotherapy: A Systematic Review and Meta-Analysis

**DOI:** 10.3390/dj14090542

**Published:** 2026-08-31

**Authors:** Tomás Quintero De La Hoz, Yaskara Sanjines Peña, Fernando Quispe Ramírez, Oriana Rivera-Lozada, Joshuan J. Barboza

**Affiliations:** 1 Instituto de Postgrado e Investigación en Ciencias de la Salud, Universidad Laica “Eloy Alfaro” de Manabí, Manta 30202, Ecuador; quinterodelahoz@gmail.com; 2 Faculdade de Odontologia de Piracicaba, Universidade Estadual de Campinas, São Paulo 13414-018, Brazil; yaskitasp@gmail.com; 3 Estomatología, Universidad Privada Antenor Orrego, Trujillo 13009, Peru; fernandoaqrg@gmail.com; 4 Vicerrectorado de investigación, Universidad Señor de Sipán, Chiclayo 14002, Peru; riveraoriana@uss.edu.pe

**Keywords:** photobiomodulation, xerostomia, head and neck cancer, radiotherapy, low-level laser therapy, salivary flow, oral pain, supportive cancer care, systematic review, meta-analysis

## Abstract

**Background**: Radiotherapy-induced xerostomia is a prevalent and disabling complication in patients with head and neck cancer, leading to persistent oral pain, impaired salivary function, and substantial deterioration in quality of life. Photobiomodulation (PBM) has emerged as a supportive therapeutic strategy with potential anti-inflammatory, analgesic, and tissue-preserving effects; however, its clinical effectiveness remains incompletely defined. **Methods**: We conducted a systematic review and meta-analysis of randomized controlled trials evaluating PBM for the management of radiotherapy-induced xerostomia in adults with head and neck cancer. MEDLINE (PubMed), Embase, Scopus, and Web of Science were searched from inception to October 2025. Eligible studies assessed intraoral and/or extraoral PBM using predefined laser parameters and reported outcomes related to oral pain, salivary flow, or xerostomia-related quality of life. Risk of bias was assessed using the Cochrane RoB 2.0 tool. Random-effects meta-analyses were performed using the Paule–Mandel estimator. Certainty of evidence was appraised using the GRADE framework. **Results**: Seven randomized trials were included. PBM significantly reduced oral pain compared with control interventions (mean difference [MD] −0.76; 95% CI −1.33 to −0.20), with moderate heterogeneity. For stimulated and unstimulated salivary flow, pooled estimates favored PBM but did not reach statistical significance, and heterogeneity was considerable (I^2^ > 90%). Substantial variability in laser wavelength, energy density, treatment frequency, anatomical targeting, and radiation exposure of salivary glands contributed to inconsistent effects across studies. The certainty of evidence was rated as moderate for pain reduction and low to very low for salivary flow outcomes. **Conclusions**: Photobiomodulation provides a clinically meaningful reduction in radiotherapy-related oral pain and may confer symptomatic benefit in xerostomia; however, its effect on salivary flow restoration remains uncertain. Standardized PBM protocols, improved methodological rigor, and adequately powered trials are required to clarify its role in supportive care for head and neck cancer patients.

## 1. Introduction

Xerostomia is characterized by decreased salivary flow and alterations in saliva composition, reflecting impaired salivary gland function [1]. Clinically, it manifests as thick and frothy saliva, dry oral mucosa, fissured tongue dorsum, and atrophy of the filiform papillae [2]. Patients undergoing cancer therapy are particularly susceptible to salivary gland hypofunction and xerostomia as a consequence of treatment-related toxicity [3].

When major salivary glands are encompassed within the irradiated field, structural and functional damage may occur, often resulting in irreversible impairment. Radiation-induced salivary gland injury typically presents as reduced saliva secretion, leading to the subjective sensation of dry mouth (xerostomia), oral discomfort, dysgeusia, dysphagia, impaired speech, and chewing difficulties, as well as an increased risk of dental caries and oral infections. Collectively, hyposalivation and xerostomia substantially compromise nutritional status, oral health, and overall quality of life (QoL) [4].

Xerostomia following radiotherapy can be broadly categorized into acute/subacute and late forms. Acute xerostomia commonly develops during or shortly after radiotherapy and is largely driven by inflammatory responses and microvascular alterations. In contrast, late xerostomia—emerging months to years after treatment—is frequently associated with irreversible acinar cell destruction and progressive glandular fibrosis. Epidemiological evidence indicates that 40–70% of head and neck cancer survivors may experience persistent xerostomia more than one year after radiotherapy, particularly those receiving higher cumulative radiation doses to the parotid and submandibular glands. Established risk factors for late hyposalivation include total radiation dose, bilateral parotid irradiation, concurrent chemotherapy, advanced tumor stage, and reduced baseline salivary reserve [5].

Several therapeutic approaches have been proposed to manage radiation-induced xerostomia in patients with head and neck cancer. Pharmacological agents such as pilocarpine and cevimeline may stimulate residual salivary function but are frequently limited by adverse effects and variable tolerability. Saliva substitutes and mouth rinses provide only temporary symptomatic relief without addressing underlying glandular injury [6,7]. Consequently, there remains a need for interventions capable of modulating tissue damage and potentially preserving residual glandular function rather than solely alleviating symptoms.

Photobiomodulation has emerged as a promising candidate in this context. Experimental and clinical studies suggest that PBM enhances mitochondrial activity via cytochrome c oxidase activation, increases ATP production, improves microvascular perfusion, and modulates inflammatory pathways. These mechanisms may contribute not only to symptomatic relief but also to protection of residual acinar cells during radiotherapy [8]. Importantly, PBM is non-invasive and associated with a favorable safety profile, making it particularly attractive for integration into oncologic supportive care protocols.

Despite growing interest in PBM, uncertainty persists regarding its independent effectiveness in radiation-induced xerostomia. Previous reviews have often evaluated heterogeneous therapeutic modalities collectively or focused on mucositis prevention without isolating xerostomia-specific outcomes. Moreover, few quantitative syntheses have specifically examined PBM as a standalone intervention while accounting for variability in dosimetry and treatment protocols. The absence of standardized parameters further complicates interpretation of the existing literature.

Therefore, the objective of this systematic review and meta-analysis is to critically evaluate and quantify the effectiveness of photobiomodulation in improving xerostomia symptoms and salivary gland function in patients with head and neck cancer undergoing radiotherapy, while examining sources of heterogeneity and methodological limitations that may inform future protocol standardization.

## 2. Materials and Methods

The protocol of this systematic review was prospectively registered in the International Prospective Register of Systematic Reviews (PROSPERO; registration number CRD420251250325).

This systematic review was structured according to the Population, Intervention, Comparator, and Outcomes (PICO) framework. The review sought to determine whether photobiomodulation therapy improves xerostomia symptoms and salivary gland function in adult patients with head and neck cancer undergoing radiotherapy, compared with sham interventions or standard supportive care. Eligible studies therefore included randomized controlled trials evaluating photobiomodulation administered intraorally, extraorally, or through combined approaches and reporting outcomes related to xerostomia severity, stimulated or unstimulated salivary flow, pain, or quality-of-life measures. This framework guided the development of the eligibility criteria and informed the construction of the search strategy described below (Supplementary Table S1).

### 2.1. Search Strategy

A comprehensive literature search was undertaken across MEDLINE via PubMed, Embase, Scopus, and Web of Science. Databases were searched from inception to 15 October 2025. The strategy incorporated controlled vocabulary, including MeSH and Emtree terms, complemented by free-text expressions related to radiotherapy-induced xerostomia, salivary gland dysfunction, and photobiomodulation. Boolean operators and truncation symbols were applied to optimize sensitivity, and the syntax of each search was adapted to the specific structure of each database. No language restrictions were applied during the search process. All records identified and assessed at both the title/abstract screening and full-text review stages were published in English. Therefore, no studies were excluded on the basis of language, and full reproducibility of the search will be ensured by making the complete strategies available in the Supplementary Materials (Supplementary Table S2).

### 2.2. Eligibility Criteria

The review considered randomized controlled trials evaluating photobiomodulation as the primary therapeutic approach for xerostomia in adults with head and neck cancer undergoing radiotherapy. Trials were eligible if they included participants who developed xerostomia or salivary gland hypofunction attributable solely to radiotherapy, and if photobiomodulation was administered intraorally or extraorally using a clearly defined laser protocol. Comparator interventions could include placebo or sham therapy, standard oral care, pharmacological agents, or other non-laser-based supportive approaches. Studies were required to report at least one of the predefined outcomes, namely salivary flow rate, xerostomia-related quality of life, or oral pain. Studies were excluded if xerostomia resulted from conditions unrelated to radiotherapy, if photobiomodulation was only one component of a multimodal therapeutic package whose individual effect could not be isolated, or if the study design did not follow a randomized structure. Non-randomized or quasi-experimental studies were excluded from the quantitative analysis but were reviewed narratively when they provided relevant clinical context regarding photobiomodulation parameters or mechanisms.

### 2.3. Study Selection

All citations retrieved from the searches were imported into Rayyan, where duplicates were removed. Titles and abstracts were screened independently by two reviewers (T.Q.D.H. and Y.S.P.). Articles deemed potentially eligible were retrieved in full text and assessed against the predefined inclusion criteria by the same reviewers. Disagreements at either screening stage were resolved through discussion and consensus; when consensus could not be reached, a third reviewer (J.J.B.) provided adjudication. Reasons for exclusion at the full-text stage were systematically documented. The overall study selection process is presented using a PRISMA 2020 flow diagram.

### 2.4. Data Extraction

Data extraction was performed independently and in duplicate by two reviewers (T.Q.D.H. and F.Q.R.), using a standardized Excel template specifically developed for this review. Extracted information included study-level characteristics such as authorship, year of publication, country, clinical setting, sample size, and overall design features. Detailed population data were collected, including the number of participants allocated to each intervention arm, sex distribution, tumor site and stage, treatment modality, total radiotherapy dose, and prior or concomitant chemotherapy regimens. Baseline xerostomia characteristics were also extracted where reported.

Intervention-related variables focused on photobiomodulation parameters, including route of delivery, device characteristics, wavelength, power output, energy density, exposure time, number of irradiation points, frequency of sessions, and total treatment duration. Information regarding comparator interventions was collected to ensure accurate contextual interpretation of treatment effects. Outcome data included stimulated and unstimulated salivary flow rates, xerostomia-related quality-of-life scores measured using validated instruments, and pain intensity predominantly assessed through visual analog scales. Discrepancies between reviewers during data extraction were resolved through discussion and consensus.

### 2.5. Risk of Bias Assessment

The methodological quality and risk of bias of the included randomized trials were assessed independently by two reviewers using the Cochrane Risk of Bias tool version 2. This approach allowed structured evaluation of potential bias arising from the randomization process, deviations from intended interventions, missing or incomplete outcome data, the manner in which outcomes were measured, and the selection of reported results. Each domain was judged according to established criteria, and an overall judgment was assigned to each study. Disagreements were resolved through discussion until consensus was reached.

### 2.6. Data Synthesis and Statistical Analysis

When at least two studies provided data for the same outcome using comparable measurement methods, quantitative synthesis was pursued. Meta-analyses were performed using a random-effects model, applying the Paule–Mandel estimator to address between-study variance. For continuous outcomes reported on a uniform scale, treatment effects were summarized as mean differences, whereas standardized mean differences were used when measurement instruments differed across studies. Dichotomous data were summarized as risk ratios with 95% confidence intervals. Statistical heterogeneity was quantified using the I^2^ statistic and interpreted according to conventional thresholds. Prediction intervals were calculated to contextualize the expected distribution of effects in future comparable clinical settings. Sensitivity analyses were conducted by excluding studies at higher risk of bias or those reporting extreme photobiomodulation parameters, acknowledging the inherent variability of laser-based interventions. Where data availability permitted, exploratory subgroup analyses were undertaken to examine whether wavelength, delivery route, or cumulative energy dosage contributed to heterogeneity. Potential publication bias was assessed for each outcome by visual inspection of funnel plots and Egger’s test, using 10 or more studies.

Exploratory subgroup analyses were conducted when feasible to investigate potential sources of heterogeneity. Subgroups were defined a priori based on (1) laser wavelength (red spectrum ≤ 700 nm versus near-infrared > 700 nm versus combined protocols), (2) route of delivery (intraoral only versus combined intraoral and extraoral application), and (3) treatment frequency (≤2 sessions per week versus ≥3 sessions per week). When sufficient data were available, descriptive comparisons were also performed according to baseline salivary gland function and reported radiation dose to major salivary glands. Due to the limited number of trials contributing to each outcome, these analyses were considered exploratory and interpreted cautiously.

### 2.7. Registration

The protocol for this review was prospectively registered in PROSPERO before data extraction to ensure methodological transparency and avoid selective reporting. The assigned registration number was CRD420251250325.

## 3. Results

### 3.1. Selection of Studies

**Figure 1:**
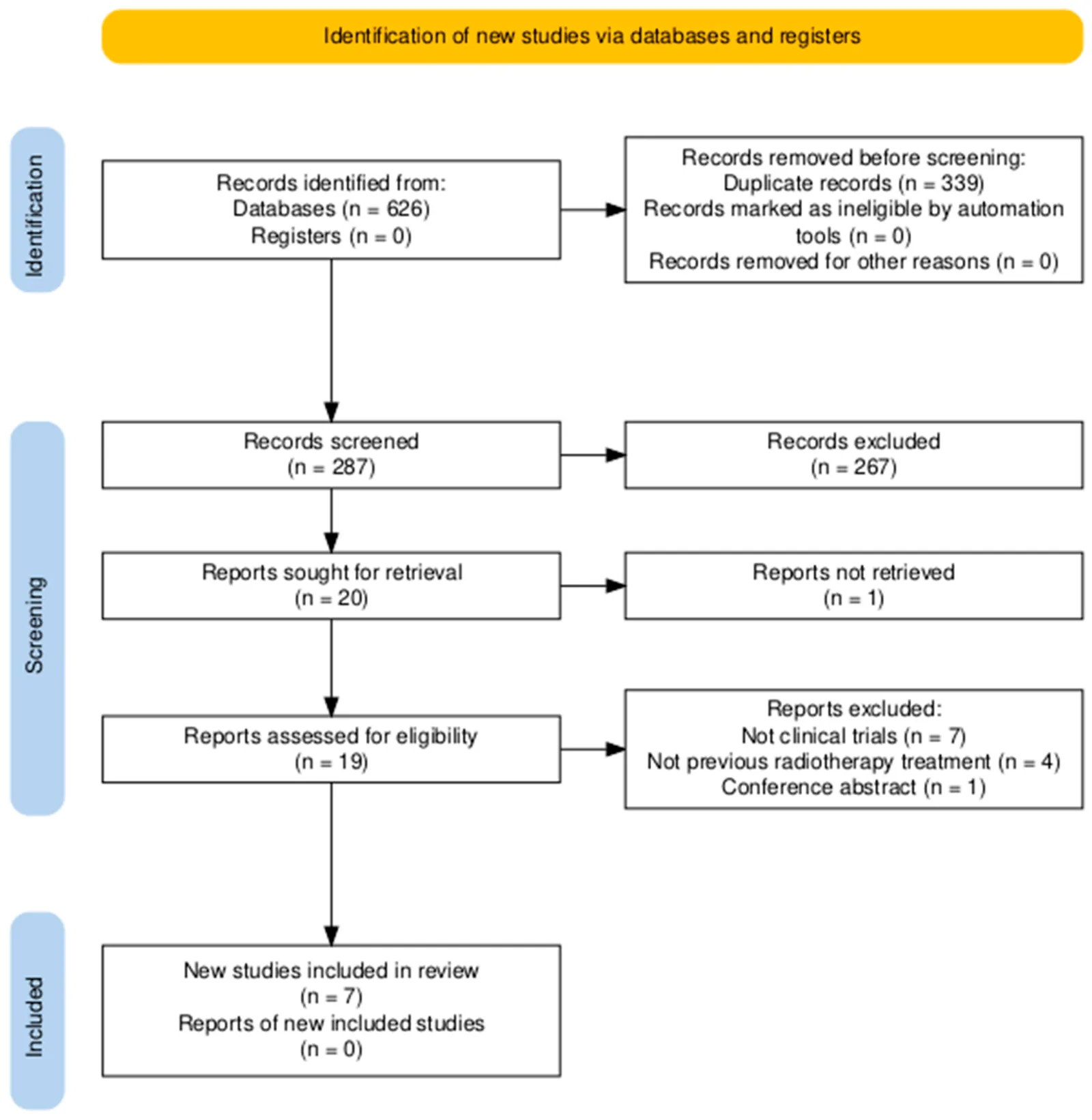
PRISMA 2020 Flow chart.

The systematic search yielded 626 records across all databases and supplementary sources (Figure 1). After removing 339 duplicate entries, 287 citations were screened by title and abstract. During this initial screening phase, 267 studies were excluded because they did not address radiotherapy-induced xerostomia, did not involve photobiomodulation as an intervention, or did not clearly meet the predefined eligibility criteria.

The full texts of 20 potentially relevant articles were then carefully assessed. Of these, studies were excluded for reasons such as xerostomia arising from mixed or non-radiotherapy etiologies, the use of photobiomodulation as part of a multimodal intervention without isolatable effects, insufficient reporting of outcomes, or a non-randomized study design. After completing the full-text evaluation, seven studies met all inclusion criteria [9,10,11,12,13,14,15].

All studies assessed at full-text stage were published in English. No eligible non-English trials were identified during the screening process.

### 3.2. Characteristics of Included Studies

The seven randomized controlled trials included in this review were conducted between 2006 and 2025, and collectively enrolled adult patients with head and neck cancer undergoing radiotherapy or chemoradiotherapy (Table 1). Most studies were performed in Brazil, with one trial conducted in Iran, reflecting a predominance of research from Latin American oncology centers where photobiomodulation (PBM) therapy is well integrated into supportive cancer care. Sample sizes varied substantially across studies, ranging from small exploratory trials with approximately 20 participants to larger trials approaching 70–80 participants per comparison group.

Across studies, the populations generally consisted of individuals receiving conventional fractionated radiotherapy for tumors located in the oral cavity, oropharynx, nasopharynx, or other head and neck sites. In most trials, xerostomia and salivary gland hypofunction were expected adverse effects given the exposure of major salivary glands to radiation fields. Some studies also allowed patients receiving concomitant chemotherapy, which is consistent with contemporary clinical practice for locally advanced head and neck cancer.

Interventions were heterogeneous in terms of wavelength, delivery route, energy parameters, and treatment schedules. Most studies used intraoral PBM protocols employing red (660–685 nm) or near-infrared (808–810 nm) wavelengths, with energy densities typically ranging between 0.7 and 2.3 J/cm^2^ per point. Several trials applied PBM two to three times per week throughout the radiotherapy course, while others compared different session frequencies (e.g., once vs. three times weekly). Two more recent studies implemented combined intraoral and extraoral PBM protocols, targeting both major and minor salivary glands. One study delivered PBM across 16 anatomical points over 4 weeks, reflecting advances in protocol standardization.

Comparator groups included sham treatments, standard oral care, lower-frequency PBM regimens, or, in one study, PBM limited solely to intraoral application. The outcomes assessed demonstrated consistency in evaluating xerostomia-related endpoints. Most trials measured unstimulated and stimulated salivary flow rates, typically collected at multiple time points during radiotherapy. Several studies incorporated validated patient-reported xerostomia scales, including visual analogue scales (VASs), the Xerostomia Inventory (XI), LENT-SOMA xerostomia grading, or quality-of-life instruments such as XeQOLS. A subset of trials also assessed xerostomia as a secondary outcome while primarily focusing on oral mucositis severity.

### 3.3. Risk of Bias Assessment

The methodological quality of the seven randomized controlled trials included in this review was evaluated using the Cochrane Risk of Bias 2.0 tool. Overall, the certainty of the evidence was influenced by recurrent issues across several domains, although one study demonstrated consistently low risk across all criteria (Table 2).

Across the domain of bias arising from the randomization process, most studies raised some concerns, primarily due to insufficient reporting of allocation sequence generation or lack of clarity regarding allocation concealment. Only three trials clearly described robust randomization procedures, resulting in a low-risk judgment. The remaining studies provided limited methodological detail, preventing a definitive assessment of whether the randomization process adequately controlled for baseline imbalances.

Regarding deviations from intended interventions, most trials were judged at low risk of bias, as interventions were protocolized, delivered by trained personnel, and generally monitored for adherence. Nevertheless, two studies raised some concerns due to incomplete descriptions of adherence monitoring, potential protocol deviations not clearly accounted for in the analysis, or absence of explicit intention-to-treat principles.

Bias due to missing outcome data varied across studies. Several trials were rated as low risk because the proportion of missing data was small and balanced between groups. However, three studies raised some concerns, often related to attrition due to radiotherapy-induced clinical deterioration or incomplete reporting of follow-up assessments. In these cases, the potential impact of missing outcomes on treatment effect estimates could not be entirely excluded.

For outcome measurement, most studies were judged as having some concerns, particularly because xerostomia and salivary flow outcomes relied on patient-reported scales or salivary collection procedures that may be susceptible to detection bias when blinding is incomplete. One study was rated at high risk due to lack of blinding of outcome assessors combined with reliance on subjective outcome measures without appropriate mitigation strategies. Only a minority of trials ensured double-blind assessment and rigorous standardization of outcome measurement.

Bias in the selection of reported results was frequently rated as some concerns. None of the included trials had publicly accessible pre-registered statistical analysis plans or detailed protocols, limiting the ability to fully exclude selective reporting. While one study provided greater transparency and was judged at low risk in this domain, reporting limitations in the remaining trials restricted confidence in the completeness and consistency of reported outcomes.

### 3.4. Effects of Photobiomodulation on Outcomes

**Figure 2:**
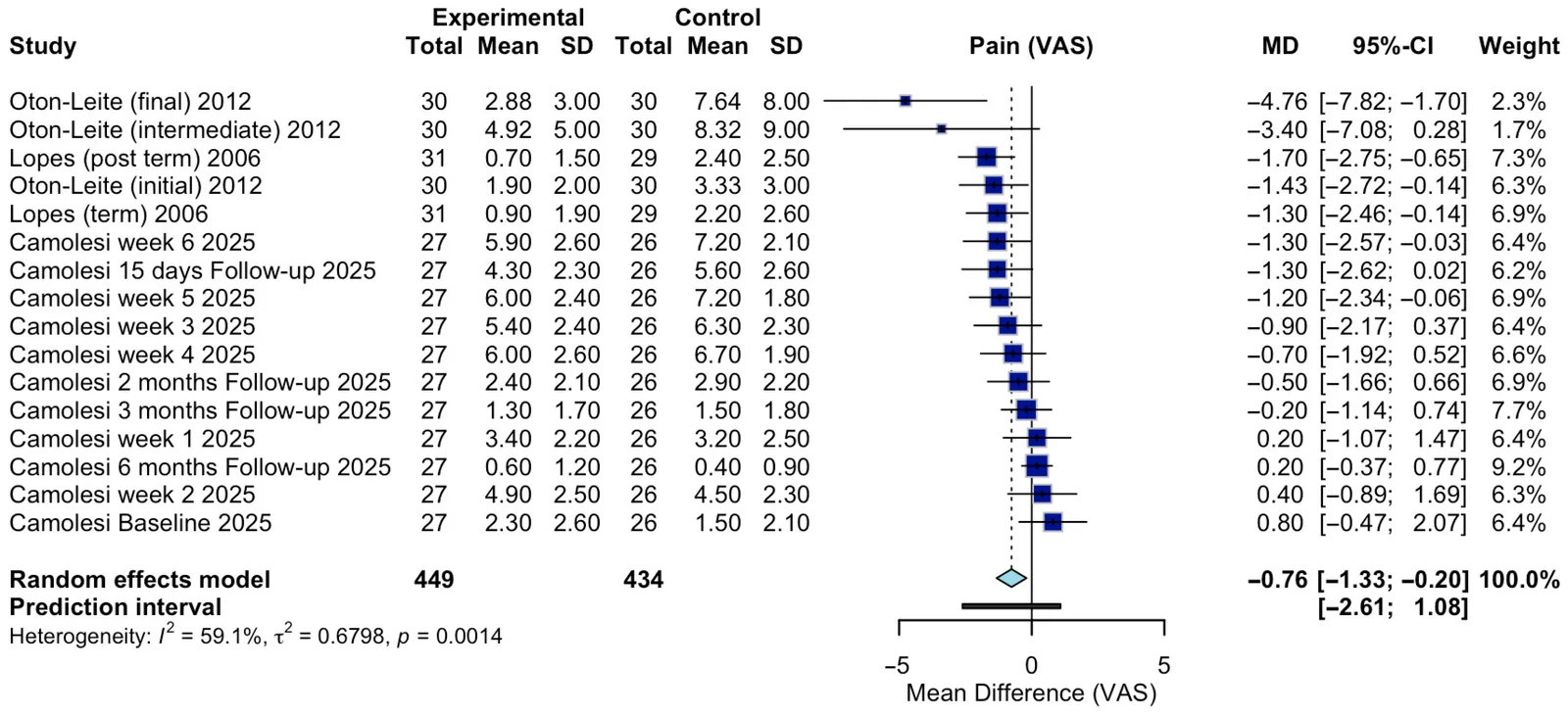
Effect of Photobiomodulation on Pain [9,10,14].

Figure 2 summarizes the effects of photobiomodulation on pain intensity measured by visual analogue scales across multiple time points from the included trials. The pooled random-effects model demonstrated a statistically significant reduction in pain favoring photobiomodulation, with a mean difference (MD) of −0.76 (95% CI: −1.33 to −0.20). Although the effect is modest, it indicates a consistent analgesic benefit compared with control conditions.

Heterogeneity was moderate (I^2^ = 59.1%), suggesting variability in effect estimates across studies, likely attributable to differences in assessment timing, laser parameters, and baseline clinical severity. The prediction interval (−2.61 to 1.08) indicates that the actual effect in future comparable populations may range from a clinically meaningful reduction in pain to negligible benefit. Overall, the evidence suggests that photobiomodulation provides a small but statistically significant decrease in radiotherapy-related oral pain.

**Figure 3:**
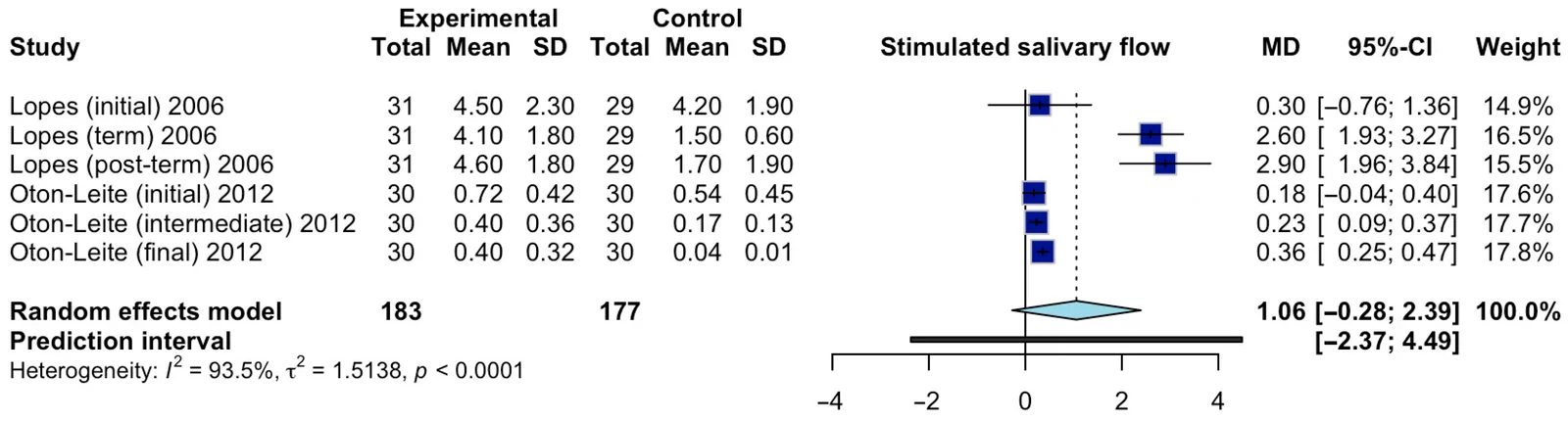
Effect of Photobiomodulation on Stimulated salivary flow [9,14].

The meta-analysis of stimulated salivary flow (Figure 3) included six comparisons from two trials that evaluated multiple time points. The pooled effect estimate showed a non-significant trend toward improved stimulated salivary flow in the photobiomodulation group, with an MD of 1.06 (95% CI: −0.28 to 2.39). Despite the direction of effect favoring photobiomodulation, the wide confidence interval indicates substantial uncertainty and precludes firm conclusions regarding efficacy.

Heterogeneity was extremely high (I^2^ = 93.5%), reflecting marked inconsistency between studies, likely influenced by differences in measurement techniques, baseline glandular function, irradiation dose, and timing of outcome collection. The prediction interval (−2.37 to 4.49) indicates that future studies may observe effects ranging from no benefit to substantial improvement, reinforcing the inherent variability in salivary flow outcomes. These findings highlight the need for standardized assessment protocols and more homogeneous laser parameters in future research.

**Figure 4:**
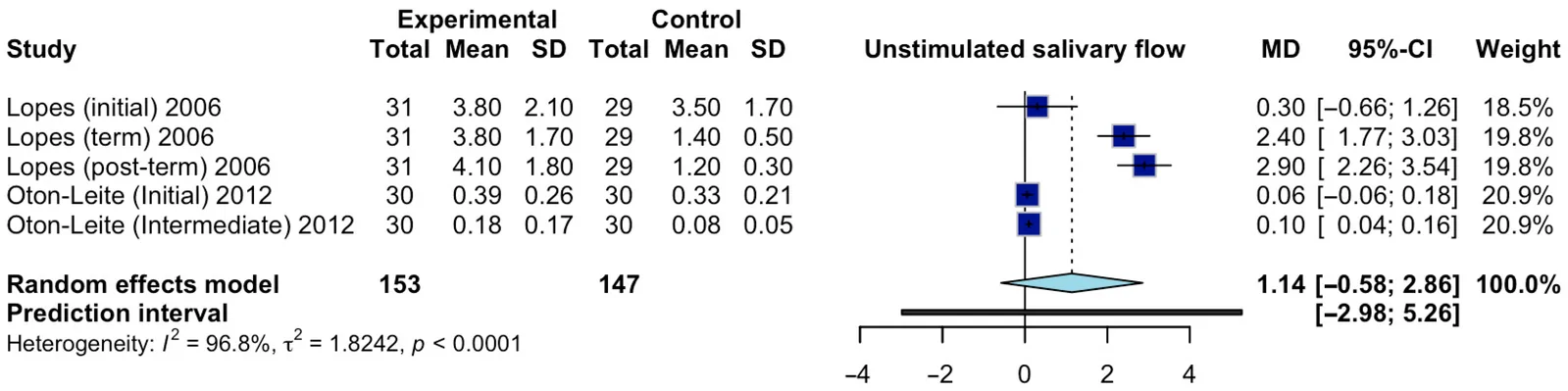
Effect of Photobiomodulation on unstimulated salivary flow [9,14].

Similarly, the pooled analysis of unstimulated salivary flow (Figure 4), based on five comparisons from two studies, showed a non-significant increase with photobiomodulation (MD = 1.14; 95% CI: −0.58 to 2.86). Although the effect estimate again favors photobiomodulation, the confidence interval crosses the null and indicates considerable imprecision.

The degree of heterogeneity was exceptionally high (I^2^ = 96.8%), underscoring pronounced divergence among studies. Differences in radiation fields, gland sensitivity, PBM dose delivery, and methodological approaches may account for this inconsistency. The prediction interval (−2.98 to 5.26) encompasses a wide range of possible actual effects, from clinically irrelevant to markedly beneficial, suggesting that unstimulated salivary flow responses to PBM are highly context-dependent.

#### 3.4.1. Exploratory Analyses of Effect Modifiers

Exploratory analyses suggested that heterogeneity in salivary flow outcomes may partially reflect differences in PBM protocols. Studies employing combined intraoral and extraoral irradiation targeting major salivary glands demonstrated numerically greater improvements in stimulated salivary flow compared with intraoral-only protocols. Similarly, trials using near-infrared wavelengths (808–810 nm), either alone or combined with red spectrum lasers, tended to report larger effect estimates than those using red light alone.

Higher treatment frequency (≥3 sessions per week) appeared to be associated with more favorable salivary outcomes, although this pattern was not consistent across all time points.

Baseline salivary gland function and radiation exposure parameters were inconsistently reported across trials, limiting formal stratified analyses. However, studies including patients with preserved baseline glandular function or lower cumulative radiation doses suggested more pronounced improvements, supporting the hypothesis that residual acinar viability may influence responsiveness to PBM.

None of the included randomized trials reported follow-up extending beyond 12 months after radiotherapy completion. Most outcome assessments were conducted during radiotherapy or within the early post-treatment phase. Consequently, the long-term sustainability of PBM effects on late xerostomia remains uncertain.

#### 3.4.2. Long-Term Follow-Up Reporting

Across the included randomized trials, outcome assessments were predominantly conducted during radiotherapy and/or within the early post-treatment period. Importantly, long-term follow-up beyond 6–12 months was not consistently reported across trials, and no trial provided sufficiently detailed long-term data to support pooled inferences on late xerostomia outcomes. Therefore, the sustainability of PBM effects on salivary gland function in the late post-radiotherapy phase cannot be determined from the currently available randomized evidence.

## 4. Discussion

The findings of this systematic review and meta-analysis demonstrate that photo-biomodulation (PBM) produces clinically meaningful improvements in pain scores and modest but directionally favorable changes in both stimulated and unstimulated salivary flow among patients with radiotherapy-induced xerostomia. In the pooled analysis for pain, PBM significantly reduced mean pain scores compared with control (MD −0.76 on a 0–10 VAS), with moderate heterogeneity across studies.

Although statistically significant, the clinical relevance of this magnitude warrants careful interpretation. In oncology and oral mucositis populations, the minimal clinically important difference (MCID) for VAS pain is commonly considered to range between 1.0 and 1.5 points. Therefore, the observed reduction, while suggestive of a measurable analgesic effect, may fall slightly below conventional thresholds for clear clinical importance. Notably, the lower bound of the confidence interval (−1.33) approaches the MCID threshold, indicating that a subset of patients may have experienced clinically meaningful benefit. Even modest reductions in pain during radiotherapy may support oral intake, treatment adherence, and overall quality of life.

In contrast, the pooled effects on stimulated and unstimulated salivary flow did not reach statistical significance, though the magnitude of effect remained consistently positive.

The clinical interpretation of these findings is strengthened by examining the underlying evidence from individual trials. Oton-Leite and colleagues reported progressive improvements in both VAS pain and salivary flow across sequential assessment points, supporting a cumulative therapeutic effect of PBM during follow-up [10]. Lopes et al. also demonstrated significant increases in stimulated and unstimulated salivary output following PBM, with larger effects observed in later post-treatment phases [9]. These findings align closely with the direction of effect observed in the present meta-analysis, particularly in early follow-up windows where glandular microcirculation and residual acinar function may still be responsive to photobiomodulatory stimuli.

More recent evidence expands this understanding. Brunelli et al. observed improvements in subjective xerostomia scores and quality of life despite variable responses in objective flow measurements, indicating that PBM’s analgesic and anti-inflammatory mechanisms may provide multidimensional benefits beyond secretory restoration [13]. Similarly, Silva et al. reported meaningful reductions in pain and oral discomfort, even in cases where salivary flow recovery was minimal, reinforcing the dissociation between symptom relief and glandular function in some patient populations [15].

In contrast, Schepanski et al. found that PBM prevented further decline in salivary function during radiotherapy but did not significantly increase post-treatment salivary flow [16]. Their biochemical analyses revealed complex shifts in salivary electrolytes and proteins, suggesting that PBM may preserve glandular integrity rather than directly stimulate secretion. This protective role may explain the heterogeneity in effect sizes across trials and aligns with the biological plausibility that severely irradiated glands with irreversible acinar loss may not regain secretory function despite improved vascularity and reduced inflammation.

The heterogeneity observed in the present quantitative synthesis likely arises from several clinical and methodological differences among included studies. Variability in laser wavelength, energy density, number of application points, frequency of sessions, and anatomical targeting may influence treatment responsiveness. For example, Oton-Leite used both intraoral and extraoral points, with progressive adjustments across time points [10], whereas Lopes employed standardized protocols across all assessments [9]. More contemporary studies, such as those by Palma et al., used high-frequency protocols over extended periods, resulting in robust improvements in salivary flow and pH [17]. These differences underscore the absence of consensus regarding optimal PBM parameters, a limitation acknowledged repeatedly in the literature.

The substantial heterogeneity observed in salivary flow outcomes is likely multifactorial. Exploratory analyses indicated that wavelength selection, anatomical targeting, and treatment frequency may act as effect modifiers. Protocols combining intraoral and extraoral irradiation of major salivary glands and those using near-infrared wavelengths appeared to demonstrate larger numerical effects. These findings are biologically plausible, as near-infrared light exhibits deeper tissue penetration, potentially enhancing stimulation of residual glandular parenchyma.

A further key limitation is the scarcity of long-term randomized follow-up. While PBM may plausibly improve symptoms during the acute/subacute phase—through anti-inflammatory and microvascular mechanisms—late xerostomia is frequently driven by progressive fibrosis and irreversible acinar loss. Because long-term follow-up beyond 6–12 months was not consistently available in the included trials, the extent to which PBM can prevent or mitigate late xerostomia, or preserve salivary gland function over time, remains uncertain. Future trials should incorporate late xerostomia endpoints (≥12 months) and standardized radiation dosimetry reporting for major salivary glands.

Furthermore, baseline salivary gland reserve and radiation dose to the parotid and submandibular glands may critically determine responsiveness to PBM. Patients with severe radiation-induced acinar destruction may derive symptomatic relief without measurable restoration of salivary flow. The inconsistent reporting of glandular radiation dosimetry across trials limited formal quantitative assessment of this hypothesis and highlights an important gap for future research.

Another key source of heterogeneity relates to patient-specific characteristics. Tumor site, total radiation dose, glandular volume encompassed within the radiation field, chemotherapy co-administration, and baseline salivary reserve capacity all influence post-RT glandular recovery. Several studies included older adults with advanced-stage cancers and high cumulative radiation exposures, potentially limiting salivary recovery regardless of intervention [14,18]. Conversely, trials enrolling patients earlier in the post-treatment trajectory or with lower radiation doses demonstrated greater improvements, suggesting a time-sensitive therapeutic window.

From a mechanistic standpoint, PBM’s benefits may derive from enhanced mitochondrial metabolism through cytochrome c oxidase activation, increased ATP synthesis, modulation of reactive oxygen species, and regulation of inflammatory cytokines, all of which may contribute to improved microvascular perfusion and tissue repair [19,20,21]. Experimental models have demonstrated that low-level laser therapy can reduce COX-2 expression and attenuate inflammatory cascades, thereby preserving tissue integrity under stress conditions [19]. Additionally, emerging evidence suggests that PBM may influence salivary cytokine profiles and epithelial homeostasis, supporting its potential role in maintaining residual acinar cell function following radiation exposure [20,21].

The high degree of heterogeneity observed in salivary flow outcomes is not merely a statistical artifact but reflects a broader methodological challenge within the field. The absence of standardized PBM protocols—including wavelength selection, energy density, irradiation points, frequency, and cumulative dose—limits the comparability of trials and hinders the development of evidence-based consensus recommendations. Without harmonized reporting and protocol standardization, it remains difficult to determine whether inconsistent findings regarding glandular flow restoration are attributable to biological variability or suboptimal treatment parameters. Establishing consensus-driven PBM dosimetry guidelines will be essential to reduce heterogeneity and improve reproducibility in future research.

This work has limitations that must be acknowledged. The number of eligible randomized trials remains small, limiting statistical power in some analyses. Heterogeneity in PBM parameters and patient characteristics complicates quantitative synthesis and interpretation. The absence of standardized laser dosimetry protocols further restricts comparability and may obscure accurate effect magnitudes. Additionally, the risk of bias varied across studies, particularly regarding allocation concealment and blinding, potentially influencing subjective outcomes such as pain and xerostomia scores.

Future research should prioritize the development of standardized PBM protocols, the evaluation of dose–response relationships, and the exploration of biological correlates of glandular responsiveness. Longitudinal studies examining durable effects beyond the acute post-treatment period are needed to clarify whether PBM offers long-term salivary rehabilitation or primarily symptomatic relief. Integration of imaging biomarkers, salivary proteomics, and machine-learning models for patient-level prediction of PBM responsiveness may further guide personalized therapeutic strategies.

Long-term Xerostomia and Sustainability of PBM Effects

An important consideration when interpreting these findings is the distinction between acute/subacute and late xerostomia. Most trials included in this review evaluated outcomes during radiotherapy or shortly thereafter, corresponding primarily to the inflammatory and potentially reversible phase of glandular dysfunction. However, late xerostomia, characterized by progressive fibrosis and irreversible acinar loss, may persist in a substantial proportion of survivors.

Recent multicentric cross-sectional evidence by Abou-Bakr et al. (2025) reported a high prevalence of persistent xerostomia and hyposalivation among long-term head and neck cancer survivors, with significant associations observed for higher cumulative radiation dose to major salivary glands, bilateral gland exposure, and concurrent chemoradiotherapy [5]. These findings highlight the chronic burden of late xerostomia and underscore the need for therapeutic strategies capable of modifying long-term glandular outcomes.

In addition to the variability observed in photobiomodulation protocols, similar methodological challenges have been reported in other areas of dental therapeutic research, where differences in procedural techniques and clinical parameters can substantially influence pooled outcomes. For instance, a recent meta-analysis comparing the tunnel technique and coronally advanced flap for the treatment of multiple gingival recession defects demonstrated that heterogeneity in surgical approaches, biomaterial use, and follow-up duration may significantly affect the magnitude and consistency of treatment effects across studies. These findings reinforce the broader methodological challenge in dental and oral medicine research of achieving standardized intervention protocols capable of producing reproducible clinical outcomes. In the context of photobiomodulation for radiation-induced xerostomia, the absence of consensus regarding wavelength selection, irradiation points, energy density, and treatment frequency likely contributes to the substantial heterogeneity observed in salivary flow outcomes [22]. Therefore, the development of consensus-driven treatment parameters and standardized reporting frameworks will be essential to improve comparability across trials and facilitate more robust quantitative synthesis in future systematic reviews.

Given that none of the included randomized trials extended follow-up beyond 12 months, the capacity of photobiomodulation to prevent or mitigate late irreversible xerostomia remains undetermined. Future randomized trials should incorporate long-term endpoints and standardized dosimetric reporting to clarify whether PBM confers durable glandular preservation or primarily short-term symptomatic relief.

Future randomized trials should prioritize late xerostomia (≥12 months post-radiotherapy) as a predefined primary endpoint, given its substantial and persistent impact on quality of life. While short-term symptomatic improvement is clinically relevant, the ultimate therapeutic goal should include long-term preservation of glandular structure and function. Incorporating standardized radiation dosimetry data, baseline salivary reserve stratification, and extended follow-up periods will be critical to determine whether PBM can meaningfully alter the trajectory of late radiation-induced glandular fibrosis.

## 5. Conclusions

In conclusion, this systematic review and meta-analysis indicates that photobiomodulation significantly reduces pain and may contribute to improvements in salivary flow among patients with radiotherapy-induced xerostomia. Nevertheless, the effects on salivary flow remain heterogeneous and should be interpreted with caution. Overall, PBM represents a biologically plausible and patient-centered supportive intervention with potential clinical value. Its integration into routine oncologic supportive care may enhance symptom control and quality of life, particularly when implemented early and delivered using standardized and evidence-informed parameters.

## Figures and Tables

**Table 1:** Characteristics of included studies.

Study	Country	Population	Sample (PBM vs. Control)	PBM Protocol (Wavelength Dose Schedule)	Comparator	Xerostomia/Salivary Outcomes	Follow-Up Reported (Duration)	Main Finding
Lopes 2006 [9]	Brazil	HNC under RT	31 vs. 29	685 nm; ≈2.3 J/cm^2^; intraoral; several sessions/week during RT	Standard care	Unstimulated & stimulated flow	During RT only; no post-RT long-term follow-up reported	PBM preserved salivary flow better than control
Oton-Leite 2012 [10]	Brazil	HNC under RT/CRT	~30 vs. ~30	685 nm; 0.8 J/point; 30 J/cm^2^ cumulative; intraoral; multiple points during RT	Sham	Unstimulated & stimulated flow	Assessed up to 30th RT session (~6 weeks); no long-term follow-up	Trends toward better preservation; not consistently significant
Louzeiro 2020 [11]	Brazil	HNC in RT	10 vs. 11	810 nm (major glands); 660 nm (minor glands); 0.7 J/point; 3×/week	Sham	Unstimulated & stimulated flow; Xerostomia VAS	During RT only; no follow-up beyond RT reported	No significant effect on flow or xerostomia
Mosannen 2024 [12]	Iran	HNC in RT	17 vs. 20	810 nm; 0.8 J/point; 2.85 J/cm^2^; 16 points; 3×/week × 4 weeks	Sham	Weekly flow; LENT-SOMA xerostomia	4–6 weeks during RT; no ≥6-month follow-up	Improved stimulated flow and lower xerostomia scores weeks 4–6
Brunelli 2025 [13]	Brazil	HNC in RT/CRT	35 vs. 35	Intraoral 660 nm (1 J/point); Extraoral 808 nm (1 J/point); multiple points	Intraoral PBM only	Salivary flow; XeQOLS	During RT; no long-term follow-up explicitly reported	Combined intra + extraoral PBM superior to intraoral alone
Camolesi 2025 [14]	Brazil	Chemotherapy recipients	27 vs. 26	660 nm intraoral + 808 nm extraoral; 1–2 J/point; 5×/week	Sham	Xerostomia symptoms (secondary)	During treatment only; no long-term xerostomia follow-up	PBM reduced symptom severity (secondary outcome)
Silva 2023 [15]	Brazil	Cancer pts in RT/CT	26 vs. 27	660 nm intraoral + 808 nm extraoral; weekly during RT	Sham	Xerostomia QoL; salivary flow	Mean 5 months (range 1–12 months); no stratified ≥12-month analysis	Improved xerostomia QoL; no significant flow change

**Table 2:** Risk of bias assessment.

Study (First Author, Year)	D1. Bias Arising from the Randomization Process	D2. Bias Due to Deviations from Intended Interventions	D3. Bias Due to Missing Outcome Data	D4. Bias in Measurement of the Outcome	D5. Bias in Selection of the Reported Result	Overall Risk of Bias
Brunelli et al., 2025 [13]	Low risk	Low risk	Low risk	Low risk	Some concerns	Some concerns
Camolesi et al., 2025 [14]	Low risk	Some concerns	Low risk	High risk	Some concerns	High risk
Mosannen Mozaffari et al., 2024 [12]	Some concerns	Low risk	Some concerns	Some concerns	Some concerns	Some concerns
Louzeiro et al., 2020 [11]	Some concerns	Low risk	Low risk	Some concerns	Some concerns	Some concerns
Oton-Leite et al., 2012 [10]	Some concerns	Low risk	Low risk	Low risk	Some concerns	Some concerns
Lopes et al., 2006 [9]	Some concerns	Some concerns	Some concerns	Some concerns	Some concerns	Some concerns

## Data Availability

Data are contained within the article.

## Supplementary Materials

The following supporting information can be downloaded at: https://www.mdpi.com/article/10.3390/dj14090542/s1, Table S1: PRISMA 2020 Main Checklist. Table S2: Search strategy for each database.
