# Supplementary material for: Effectiveness of Photobiomodulation as a Treatment for Xerostomia in Patients with Head and Neck Cancer Undergoing Radiotherapy: A Systematic Review and Meta-Analysis
Source: Dent J (Basel). 2026 Aug 31;14(9):542. doi: 10.3390/dj14090542 (PMC13605498; doi:10.3390/dj14090542)
Supplement: Supplementary file 1 [file dentistry-14-00542-s001.zip › dentistry-4125690-supplementary.pdf]

Supplementary table S1. Search strategy for each database

Engine	Strategy	Results
**Pubmed**	("radiotherapy-induced xerostomia" OR "radiotherapy induced xerostomia" OR xerostomia OR asialia OR “mouth dryness” OR “dryness, mouth” OR hyposalivation OR hyposalivations)	82
	(“Light Therapies, Low-Level” OR “Light Therapy, Low-Level” OR “Low-Level Light Therapies” OR “Low Level Light Therapy” OR “Therapies, Low-Level Light” OR “Therapy, Low-Level Light” OR “LLLT” OR “Photobiomodulation Therapy” OR “Photobiomodulation Therapies” OR “Therapies, Photobiomodulation” OR “Therapy, Photobiomodulation” OR “Photobiomodulation” OR “Photobiomodulations” OR “Laser Therapy, Low-Level” OR “Laser Therapies, Low-Level” OR “Laser Therapy, Low Level” OR “Low-Level Laser Therapies” OR “Laser Biostimulation” OR “Biostimulation, Laser” OR “Laser Irradiation, Low-Power” OR “Irradiation, Low-Power Laser” OR “Laser Irradiation, Low Power” OR “Laser Phototherapy” OR “Phototherapy, Laser” OR “Laser Therapy, Low-Power” OR “Laser Therapies, Low-Power” OR “Laser Therapy, Low Power” OR “Low-Power Laser Therapies” OR “Low-Level Laser Therapy” OR “Low Level Laser Therapy” OR “Low-Power Laser Irradiation” OR “Low Power Laser Irradiation” OR “Low-Power Laser Therapy” OR “Low Power Laser Therapy”)	
**Scopus**	TITLE-ABS-KEY("radiotherapy-induced xerostomia" OR "radiotherapy induced xerostomia" OR xerostomia OR asialia OR “mouth dryness” OR “dryness, mouth” OR hyposalivation OR hyposalivations)	225
	TITLE-ABS-KEY(“Light Therapies, Low-Level” OR “Light Therapy, Low-Level” OR “Low-Level Light Therapies” OR “Low Level Light Therapy” OR “Therapies, Low-Level Light” OR “Therapy, Low-Level Light” OR “LLLT” OR “Photobiomodulation Therapy” OR “Photobiomodulation Therapies” OR “Therapies, Photobiomodulation” OR “Therapy, Photobiomodulation” OR “Photobiomodulation” OR “Photobiomodulations” OR “Laser Therapy, Low-Level” OR “Laser Therapies, Low-Level” OR “Laser Therapy, Low Level” OR “Low-Level Laser Therapies” OR “Laser Biostimulation” OR “Biostimulation, Laser” OR “Laser Irradiation, Low-Power” OR “Irradiation, Low-Power Laser” OR “Laser Irradiation, Low Power” OR “Laser Phototherapy” OR “Phototherapy, Laser” OR “Laser Therapy, Low-Power” OR “Laser Therapies, Low-Power” OR “Laser Therapy, Low Power” OR “Low-Power Laser Therapies” OR “Low-Level Laser Therapy” OR “Low Level Laser Therapy” OR “Low-Power Laser Irradiation” OR “Low Power Laser Irradiation” OR “Low-Power Laser Therapy” OR “Low Power Laser Therapy”)	

**Web of Science**	("radiotherapy-induced xerostomia" OR "radiotherapy induced xerostomia" OR xerostomia OR asialia OR “mouth dryness” OR “dryness, mouth” OR hyposalivation OR hyposalivations)	75
	(“Light Therapies, Low-Level” OR “Light Therapy, Low-Level” OR “Low-Level Light Therapies” OR “Low Level Light Therapy” OR “Therapies, Low-Level Light” OR “Therapy, Low-Level Light” OR “LLLT” OR “Photobiomodulation Therapy” OR “Photobiomodulation Therapies” OR “Therapies, Photobiomodulation” OR “Therapy, Photobiomodulation” OR “Photobiomodulation” OR “Photobiomodulations” OR “Laser Therapy, Low-Level” OR “Laser Therapies, Low-Level” OR “Laser Therapy, Low Level” OR “Low-Level Laser Therapies” OR “Laser Biostimulation” OR “Biostimulation, Laser” OR “Laser Irradiation, Low-Power” OR “Irradiation, Low-Power Laser” OR “Laser Irradiation, Low Power” OR “Laser Phototherapy” OR “Phototherapy, Laser” OR “Laser Therapy, Low-Power” OR “Laser Therapies, Low-Power” OR “Laser Therapy, Low Power” OR “Low-Power Laser Therapies” OR “Low-Level Laser Therapy” OR “Low Level Laser Therapy” OR “Low-Power Laser Irradiation” OR “Low Power Laser Irradiation” OR “Low-Power Laser Therapy” OR “Low Power Laser Therapy”)	
**Embase**	"radiotherapy-induced xerostomia"	69
	(“Low Level Light Therapy” OR “LLLT” OR “Photobiomodulation Therapy”)	
	TOTAL SEARCH	626
	DUPLICATES	339
	TOTAL REGISTERS AFTER DUPLICATES REMOVED	287
